# Adherence to the Mediterranean Diet in a School Population in the Principality of Asturias (Spain): Relationship with Physical Activity and Body Weight

**DOI:** 10.3390/nu13051507

**Published:** 2021-04-29

**Authors:** Rocío Fernández-Iglesias, Sonia Álvarez-Pereira, Adonina Tardón, Benjamín Fernández-García, Eduardo Iglesias-Gutiérrez

**Affiliations:** 1 Spanish Consortium for Research on Epidemiology and Public Health (CIBERESP), Monforte de Lemos Avenue, 3-5, 28029 Madrid, Spain; atardon@uniovi.es; 2 Department of Medicine, Unit of Molecular Cancer Epidemiology, University Institute of Oncology of the Principality of Asturias (IOUPA), University of Oviedo, Julian Clavería Street s/n, 33006 Oviedo, Spain; 3 Instituto de Investigación Sanitaria del Principado de Asturias (ISPA), Roma Avenue s/n, 33001 Oviedo, Spain; fernandezbenjamin@uniovi.es (B.F.-G.); iglesiaseduardo@uniovi.es (E.I.-G.); 4 Department of Morphology and Cell Biology, University of Oviedo, Julian Clavería Street s/n, 33006 Oviedo, Spain; uo217156@uniovi.es; 5 Department of Functional Biology, University of Oviedo, Julian Clavería Street s/n, 33006 Oviedo, Spain

**Keywords:** Mediterranean diet, dietary habits, children, KIDMED, Principality of Asturias, lifestyle, obesity

## Abstract

The Mediterranean diet (MD), despite its multiple benefits, presents low levels of adherence among children. Moreover, childhood is a key stage in the acquisition of healthy habits. The aim of this study was to describe adherence to MD in school-age children from Asturias, Spain, and to evaluate the association with weight status and several lifestyle behaviors. A cross-sectional study was conducted on 309 children aged between 8 and 13 years old. The level of adherence to MD was evaluated through the KIDMED questionnaire. Descriptive analysis and logistic regression models were used to analyze the association between adherence to MD and weight status, frequency of out-of-school exercise, frequency of school canteen attendance, and sleep habits. We found that 54.4% of children had optimal adherence to MD and 29.9% of the sample was overweight or obese. Frequency of exercise practice was positively associated with optimal adherence to MD (95% CI: 1.02, 1.33). A positive association was found between some KIDMED items and frequency of out-of-school exercise practice and attendance at the school canteen. This study shows the need for an improvement in the adherence to MD in youth considering the concomitant occurrence of other related healthy behaviors.

## 1. Introduction

Children overweight and obesity has become an increasing worldwide health problem in the last few decades. Globally, the prevalence of overweight and obesity among children aged 5–19 has risen dramatically from just 4% in 1975 to over 18% in 2016 [1]. This increasing prevalence is associated with the emergence of comorbidities previously considered to be “adult” diseases, including type 2 diabetes mellitus, hypertension, nonalcoholic fatty liver disease, obstructive sleep apnea, and dyslipidemia [2], as well as contributing to behavioral and emotional complications in children [3]. In Europe, a high prevalence of overweight among children has been observed in western and especially southern countries, with England, Greece, Italy, Malta, and Spain at the top [4]. Regarding Spain, the prevalence of children who are overweight and obese is 13.9% and 12.4%, respectively (26.3% combined), and they are more common in males than in females (15.6% and 12%, respectively, for obesity). A high prevalence is found among those aged between 6 and 13 years of age, and significant differences are also evident between geographic areas [5]. The Principality of Asturias, a region located on the northern coast of Spain, has an alarming prevalence of children who are overweight and obese (33.3%), with a higher prevalence of obesity in males than in females (12.4% and 9.2%, respectively) and the highest rate in children between 7 and 14 years, according to the ESNUPI-AS study [6].

Childhood obesity is a complex and multifactorial disease. Research suggests that children are frequently exposed to an unhealthy environment that increases the risk of suffering obesity through a combination of multiple risk factors [3,7]. Dietary patterns are strong determinants of overweight and obesity in children and adolescents [2,8], although low levels of physical activity [9,10] and less sleeping time [11,12] have been described as negative correlates with a healthy body weight.

Regarding the influence of dietary habits, there is evidence that energy-dense diets, high in saturated-fat and with a low-fiber content, predispose young people to later overweightness and obesity [8]. Furthermore, the consumption of added sugars, with sweet products and sweetened beverages as the major contributors [13,14], has also been associated with weight gain and insulin resistance [15,16,17]. Westernized dietary habits, which include an increase in energy intake, a higher consumption of meat and foods with low nutritional density, and a decreased consumption of fruit and vegetables, have been adopted by the young population [18,19]. Other than these, there are dietary patterns whose health benefits have been corroborated by numerous epidemiological and experimental studies, such as the Mediterranean diet (MD) [20,21]. This cultural dietary model is based on the high consumption of extra virgin olive oil (as the main source of added fat), vegetables, fruits, cereals, legumes, nuts and seeds, moderate intakes of fish, seafood and other meat, dairy products, and low intakes of eggs, sweets, and highly processed foods [22]. A wide range of studies have shown that a higher adherence to the MD is associated with a reduced risk of overall mortality, cardiovascular diseases, cancer incidence, mental and neurodegenerative diseases, diabetes and coronary heart disease [20,23,24,25,26,27]. Furthermore, other studies suggest that adherence to the MD is associated with lower abdominal adiposity and reduced weight gain [24,25], with a beneficial effect on anthripometric parameters and cardiometabolic risk factors. Moreover, MD has social and environmental benefits, centered on sustainability [28,29].

In spite of all these benefits, the adherence to the MD is decreasing among children and adolescents [30,31,32,33]. In Spain, the enKid study showed that only 46.4% of children had an optimal adherence to the MD, although this percentage differed between regions [33,34].

Based on the above findings, evaluating the level of adherence to MD, as well as the associated and interacting lifestyle factors, becomes essential for designing public health interventions that can contribute to the acquisition of healthy lifestyle habits by children, with consequent impacts on the control and prevention of obesity and cardiovascular diseases in childhood and later life [35].

Therefore, this study aimed to describe adherence to the MD in school-age children from the Principality of Asturias (Spain), as well as to evaluate the association with weight status and other lifestyle behaviors.

## 2. Materials and Methods

### 2.1. Participants and Sampling

This was a cross-sectional study aimed at children in the third and sixth grades of Primary School in the Principality of Asturias (Spain).

For the selection of the sample, 12 school centers were randomly selected to cover the three main areas of the Principality of Asturias (central, east and west) and rural and urban settings. The number of centers in rural and urban settings in each area was the same. The cut-off point to define a rural or urban setting was established at 10,000 inhabitants [36], and demographic data were obtained from the 2017 SADEI census [37]. Of the 12 centers that were asked to participate (389 children were potential participants), 10 accepted (83.3%).

**Figure 1:**
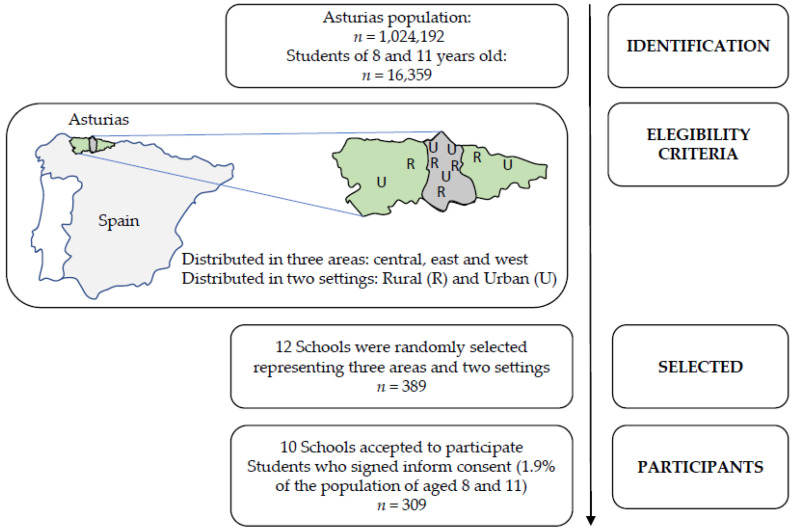
Flowchart of the sample selection process.

In these 10 centers, a total of 309 (79.4%) students took part, 151 girls and 158 boys (48.9% and 51.1%, respectively). Of these 309 children, 138 were in third grade and 171 in sixth grade (44.7% and 55.3%, respectively). Therefore, the study population age was between 8 and 13 years (Figure 1). The age of the participants was chosen, taking into account that the latest studies show a high prevalence of overweight and obesity in this age group in the Principality of Asturias [6]. Furthermore, children at those ages have sufficient autonomy to be able to complete the questionnaire by themselves, under the supervision of their teachers and the researchers involved in the study.

### 2.2. Data Collection Instruments

Data collection took place in 2018 by means of an ad hoc questionnaire made up of three parts: (i) personal data and anthropometric measurements; (ii) KIDMED test, food intolerances, and school canteen attendance, and (iii) other lifestyle behaviors (sleeping habits and physical activity habits outside of school).

Children’s age, gender, and school educational level, all of them previously related to the adherence to the MD in several studies [10,33,38,39,40], were recorded. Other variables related to adherence to the MD, such as parental educational level [41,42,43] and the school’s area and setting [30,38,40], were also recorded. The collection of these variables allows us to control them as possible confounding variables.

Regarding anthropometric measurements, weight and height were self-reported by children. BMI ($weight/height^{2}$) z-scores were calculated using age- and sex-specific reference values from the International Obesity Task Force [44,45], giving rise to a qualitative variable formed from the following categories: underweight, normal-weight, overweight, and obesity.

The adherence to the MD was assessed through the Mediterranean diet quality index, KIDMED, a 16-item questionnaire designed for children and youth, as described elsewhere [34]. The overall score can range from −4 to 12, and is categorized into three levels: ≤3 low, 4–7 medium, and ≥8 optimal adherence [34].

With respect to other eating patterns and lifestyle behaviors, the number of days the students attended the school canteen and the presence/absence of food intolerances were recorded. Children were also asked about the number of hours they regularly sleep on weekdays and the number of hours a week they practice exercise outside of school.

### 2.3. Data Collection Procedure

This project was evaluated, approved, and registered (Code: CEImPA 2021.245) by the Ethical Committee of the Hospital Universitario Central of Asturias (CEIM). All enrolled schools received an email to be forwarded to the families carefully explaining the purpose, the protocol and the methods of the study, the role of the school in it, the corresponding questionnaire, and a letter of informed consent for parents and children to sign.

In order to carry out the data collection process, a member of the research team attended the centers by appointment. All the questionnaire items were self-reported by the children, with help and supervision from the examiner and teachers.

### 2.4. Statistical Analysis

Descriptive statistics were calculated for the final sample using frequencies and percentages for qualitative variables, and using mean and standard deviation (SD) for quantitative variables. To carry out bivariate analysis, differences between continuous variables were evaluated using Pearson and Spearman correlation coefficients, depending on the distribution of the data. Differences between categorical variables were evaluated by Chi-squared or Fisher exact test, as appropriate, and effect sizes were obtained using Cramer’s V measure, considering the effect size as trivial (<0.1), weak (0.1–0.3), moderate (0.3–0.5) or strong (>0.5). Finally, differences between continuous and categorical variables were analyzed by t-Student or ANOVA test for normally distributed continuous variables, and by Mann–Whitney or Kruskal–Wallis tests for not normally distributed continuous variables.

Univariate and multivariate logistic regression models were created in order to analyze and quantify the association between adherence to the MD (treated as the dependent variable) and children BMI, exercise practice outside school, school canteen attendance and sleeping habits (as the independent variables). As the prevalence of a low adherence to the MD was relatively low, medium and low categories were considered together in the final analysis, resulting in a dichotomous variable. The BMI variable was transformed the same way, by unifying the overweight and obesity categories into one, and underweight and normal categories into another. Bivariate analysis was used to identify the variables related to both dependent and independent variables, in order to consider those with *p*-value < 0.2 as potential confounders, from among those selected from the literature. To avoid overfitting, only those confounding variables that modified the independent coefficient by >|10%| following forward stepwise were included in the final model. The same analysis was run considering each one of the KIDMED items as the dependent variable in the models.

For the sensitivity analyses, we repeated the analysis using BMI as a continuous variable, as well as recalculating BMI z-scores using WHO reference values [46]. We ran the analyses with all the independent variables included simultaneously in the model to obtain independent associations between each lifestyle factor and the adherence to the MD. We also repeated the analyses, including school educational level and gender interaction terms, but these were not statistically significant in the models.

The odds ratio (OR) was estimated with 95% confidence intervals (CI) and the level of statistical significance was fixed as *p*-value < 0.05. Statistical analyses were carried out using the R 3.6.3 statistical software [47].

## 3. Results

Table 1 contains the description of the total sample characteristics. More than one-quarter of the children were overweight or obese (23.1% overweight, 95% CI: 18.6%, 28.3%; 6.8% obesity, 95% CI: 4.4%, 10.4%), without differences between boys and girls, or between school educational levels. Regarding lifestyle behaviors, there were statistically significant differences by sex in the frequency of sport practice outside school, this being higher in boys than girls in both educational levels. Third grade children tend to attend the school canteen more days than those in sixth grade.

**Figure 2:**
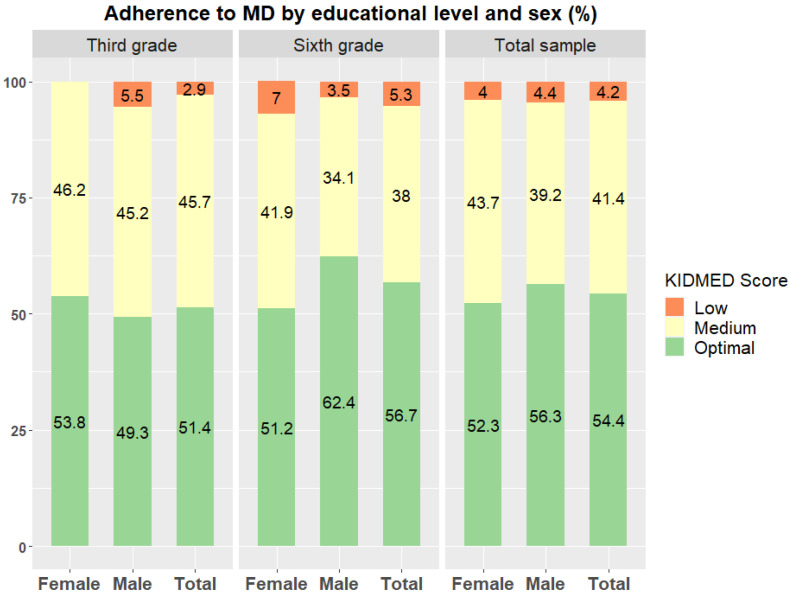
Description of level of adherence to the MD of the total sample (309) based on the KIDMED score. Data are presented as frequency (%) of children classified in each category, stratified by sex and school educational level.

The mean (SD) score obtained in the KIDMED test was 7.5 (2.2) in the total sample, with a mean of 7.5 (2.1) for girls and 7.6 (2.3) for boys. The mean score obtained in third graders was 7.6 (2.2) (7.9 (1.9) in girls and 7.3 (2.4) in boys), and the mean score obtained in sixth graders was 7.6 (2.2) (7.2 (2.1) in girls and 7.9 (2.1) in boys). No statistically significant differences were found by year group or sex. Figure 2 shows the distribution of the levels of adherence to MD according to the KIDMED score, stratified by school educational level and sex. Most children showed an optimal adherence to the MD, with no differences between school educational level or between boys and girls. Although less than 7.3% of the children analyzed showed low adherence to MD, more than 34.6% showed a sub-optimal adherence. In the total sample, the distribution of the levels of adherence was as follows: 4.2% low adherence (95% CI: 2.4%, 7.3%), 41.4% medium adherence (95% CI: 35.9%, 47.2%) and 54.4% optimal adherence (95% CI: 48.6%, 60.0%). Furthermore, no differences in optimal adherence to the MD were observed by other factors, such as rural or urban environment or parental educational level.

**Figure 3:**
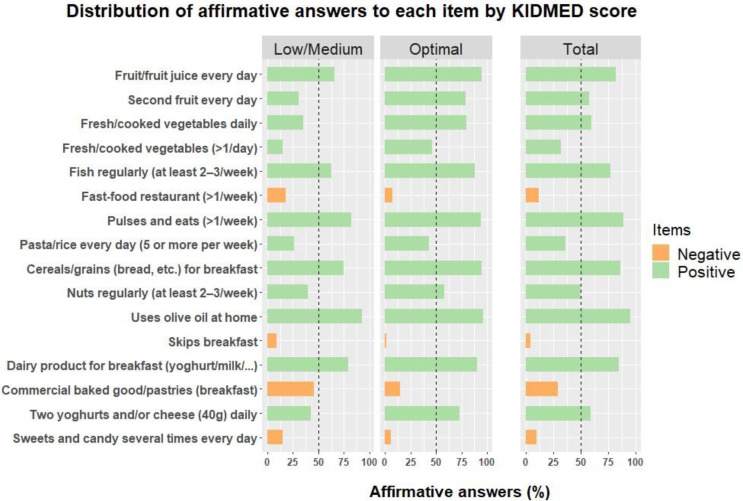
Percentage of affirmative responses of the total sample (309) to each item of the KIDMED test. The results are presented aggregated (Total sample) and stratified by the categories of the KIDMED test (Low/Medium vs. Optimal).

Figure 3 shows the frequency of affirmative responses to each KIDMED item by KIDMED score (classified into “low/medium” or “optimal” adherence). We have not found statistically significant differences between boys and girls. Statistically significant differences between school educational levels were only observed in pulses consumption, this being higher in third grade children. Globally, the items with the highest frequencies of response were the use of olive oil (93.9%), and cereals or grains (86.1%), pulses (87.7%) and dairy product (84.1%) consumption. Items with the lowest frequencies were mainly those associated with a negative dietary pattern: skipping breakfast (5.8%), sweets and candy consumption (10.4%), and going to fast-food restaurants (12.3%). Among those items with a negative association with MD, the highest frequency of response was observed for commercially baked goods or pastries consumption (29.1%).

In each of the KIDMED score categories, the distribution was as follows: children with low/medium adherence had a good response in terms of the consumption of olive oil (93.0%), pulses (82.2%), dairy products (79.1%), cereals or grains (75.2%), one piece of fruit per day (65.9%), and fish (62.8%). With respect to negative items, the highest frequency of affirmative responses was for commercial baked goods or pastries consumption (45.7%). In the “optimal” adherence category, all positive items had high frequencies of affirmative response, except for the consumption of vegetables more than once a day, and pasta or rice more than once a day, with less than 50% affirmative answers. The negative item with the highest frequency of affirmative responses was the consumption of industrial baked goods or pastries, with a 14.8% of affirmative answers.

The detailed results of the KIDMED test are available as Supplementary Material Table S1.

The use of olive oil is not related with KIDMED score (*p*-value = 0.4; Cramer’s V = 0.06). The items that discriminated most strongly between low/medium and optimal adherence are a fruit or fruit juice, the consumption of a second piece of fruit every day (*p*-value < 0.01, Cramer’s V = 0.38 and *p*-value < 0.01, Cramer’s V = 0.48, respectively), vegetables intake once a day or more than once a day (*p*-value < 0.01, Cramer’s V = 0.46 and *p*-value < 0.01, Cramer’s V = 0.33, respectively), fish consumption (*p*-value < 0.01, Cramer’s V = 0.30), consumption of two yoghurts and/or some cheese daily (*p*-value < 0.01, Cramer’s V = 0.31), and commercially baked goods or pastries for breakfast (*p*-value < 0.01, Cramer’s V = 0.34).

The results of the multivariate logistic regression for the KIDMED score and each of the KIDMED items as the dependent variables are given in Table 2. Using the KIDMED score as the dependent variable, we observed that those children who practiced exercise more days per week outside school were more likely to exhibit optimal adherence to the MD (OR = 1.17, 95% CI: 1.02,1.33). We did not find a statistically significant association between optimal adherence to the MD and BMI. We observed the same with the number of sleep hours on weekdays and the number of days that children attend the school canteen, although these variables had a positive OR.

Using each one of the KIDMED items as the dependent variable, we obtained the following statistically significant results: BMI categories were positively associated with the intake of dairy products for breakfast. Frequency of exercise practice outside school was a positive predictive factor for the intake of fruit or fruit juice every day, as well as for cereals or grains for breakfast.

In addition, the frequency of school canteen attendance was positively predictive for intake of fruit or fruit juice every day, and for fish. It should also be noted that, although the association was not statistically significant with vegetables intake once a day or more than once a day, the trend in this direction is clearly positive (*p*-value = 0.06 for both). Finally, we did not find a statistically significant association between the number of hours that children sleep during weekdays and any KIDMED item.

## 4. Discussion

In the present study, just over half of the school children analyzed showed an optimal adherence to MD (54.4%). Similar results have been observed in other studies carried out with Spanish and Portuguese children. In Spain, the enKid study showed an optimal adherence in 46.4% of children [34], while The Eat Mediterranean Program in Portugal reported an optimal adherence in 58.4% of the sample [43]. These results are in contrast with other Mediterranean countries, such as Greece, Cyprus, or, to a lesser extent, Italy, where lower optimal MD adherence percentages have been described. Thus, in Greece, the GRECO study showed only a 4.3% optimal adherence to the MD. Similar results have been observed in Cyprus, where the LLAL study showed 6% optimal adherence. In Italy, more variable percentages have been observed, with optimal adherence ranging from 5 to 19.6% [41,48]. By contrast, the levels of adherence to the MD in northern Europe are higher than those previously mentioned, with optimal adherence percentages of 14.3% and 24.3% [49,50]. Although the level of adherence to the MD is considerably higher in this research, all these results indicate the need for an improvement in the adherence to the MD in youth. This is particularly relevant considering that the Principality of Asturias is characterized by the lowest birth rate in Europe (5.6 births per 1000 people) [51], and it is among the seven oldest (is one of the nine regions in Europe where the decrease in population is projected to be greater than 25% by 2050) [52].

Interestingly, we have not found significant differences in optimal adherence to the MD by factors such as gender, age, school educational level, rural or urban environment, or mother’s educational level, even though it should be noted that in the case of the mother’s educational level, the results obtained may be due to the high number of missing values in the sample. Although several studies have shown these factors as predictors of MD adherence, the latest systematic reviews indicate that the results in this regard are not consistent, especially in terms of gender and age [30,40]. However, differences by gender have been found in frequency of exercise practice outside of school, this being higher in boys. This result has been observed in other studies, where less physical activity is observed in females than in males, and this decline with age [7]. Furthermore, data on physical activity in Spain, as well as in other European countries, indicate that it tends to drop off between the ages of 11 and 15. The average level of physical activity in boys decreases by 50% between the ages of 11 and 15 years, this being even more dramatic in girls [53].

This study reported positive dietary habits for fruit, fish, pulses and olive oil consumption, as well as the consumption of dairy products and cereals or grains for breakfast. Although the consumption of a second piece of fruit per day, or of vegetables once or more than once a day, should still be improved (less than 65% of the sample answered affirmatively to one of these items), the values observed in this study regarding these habits are in line with, or above, those observed in other Spanish [42,54] and Italian [11] populations. The positive habits that most determine an optimal versus a low/medium adherence in the study sample are fruit consumption (one or more a day), vegetable consumption (one or more times a day), regular fish consumption, and daily yoghurt or cheese consumption. In contrast, the negative habit that most characterizes the difference between these two groups of adherence (optimal versus medium/low) is the consumption for breakfast of commercially baked goods or pastries. In total, 14.8% of children showing optimal adherence answered affirmatively to this habit, while 45.7% of children with low or medium adherence did so. At the other extreme, the only habit that does not contribute in any way to differentiating between adherence categories is the consumption of olive oil. The high percentage of children who report they use olive oil at home (over 92% in all adherence categories) is especially relevant considering the benefits of exclusive olive oil consumption as an added fat, which was associated with metabolic indices such as obesity and cardiorespiratory fitness, and a healthy lifestyle profile [27].

The results obtained from the logistic regression analysis allowed us to identify a positive association between the number of days that children practice exercise outside of school and an optimal adherence to MD. In reference to exercise practice, the results were coincident with those observed in numerous studies [10,38,39,42,50]. This result is further supported by a systematic review of adherence to the MD in children and adolescents, which indicates that this association is consistent [40]. In our study, participation in exercise activities out of school was self-reported in terms of number of days per week, while daily physical activity or energy expenditure was not measured. Despite this, the association has been significant. This may be due to the fact other studies show that this association does not depend on the intensity [42].

Furthermore, it was observed that those children who practice exercise more days per week were more likely to consume a piece of fruit or a fruit juice every day, and to have cereals or grains for breakfast. These results are in line with recent research, which links frequent weekly physical activity to healthy choices, such as increased consumption of fruits and cereals, but also vegetables, dairy products, fish and nuts [55]. This could be explained by the healthy environment that usually surrounds the practice of sport, focused on covering the nutritional needs of active young people and taking care of their physical health. This highlights the importance of promoting participation in sports activities and the practice of physical exercise among young people, not only because of the indubitable benefits for health in the short and long terms, but also for its indirect influence on other healthy behaviors, such as food choices and nutritional intake [56,57].

On the contrary, no association has been observed between the level of adherence to MD and BMI, sleeping time, or children’s school canteen attendance. Regarding BMI, this result was in line with the literature, since most studies in which this association was evaluated have failed to find significant associations among these parameters [10,11,38,40,42,49,58]. Surprisingly, the logistic regression results showed that those children who have a dairy product for breakfast were more likely to be overweight or obese, independently of their adherence to MD. However, this is a complex association that can be biased by other factors, leading to conflicting results. In this study, the lack of information about energy intake and more detailed information about frequencies and amounts of food consumption do not allow for controlling the influence of these potential cofounders. On the other hand, the limitations of BMI as a weight status and body composition indicator have been discussed in the literature, considering other alternatives such as waist circumference, skinfold thickness, or bioelectrical impedance analysis, among others [2,59].

As regards sleep duration, some studies have found a significant association with food choices or BMI, but not specifically with adherence to the MD [11,12]. Neither have we observed a significant association between sleep duration and any KIDMED test item. This question deserves further investigation. Not using a specific and validated test to precisely measure sleep duration could be influencing this lack of association.

Regarding school canteen attendance, results showed that there was a positive relationship with the consumption of fruit every day, as well as with fish. Furthermore, although it is not statistically significant, there is a clear positive trend with the consumption of vegetables once or several times a day. These are relevant results, since they show the important role that the school environment can play in children’s dietary patterns and the acquisition of healthy habits, it being a suitable place to carry out interventions.

## 5. Limitations

Among the study limitations, we found that, due to its descriptive and cross-sectional nature, it is not possible to establish causality for the associations studied. However, the significant associations found and their concordance with other studies highlight the relevance of the results obtained and the need for further research in this line. The use of a self-reported questionnaire constitutes another limitation. This could introduce biased information, such as overreporting healthy behaviors and underreporting what they consider to be less healthy. However, the reliability of the KIDMED questionnaire has been previously verified on children [60,61], although they were older than those in this study. It should also be noted that no exclusion criteria that affect eating behaviors and diet-related diseases were considered in the selection process of potential study participants, with the consequent limitations that this may involve. Finally, due to the few subjects classified in the low-adherence category, the variable that measures adherence to the MD was dichotomous, distinguishing between “optimal” and “sub-optimal” adherence. This may diminish the resolution of the analysis in distinguishing nuances between adherence groups, although it allows an in-depth analysis adapted to the specific characteristics of the population studied.

## 6. Conclusions

This study shows a suboptimal adherence to the MD in Spanish children, which highlights the importance of implementing educational programs aiming to improve their eating habits and nutritional intake. The necessity for a holistic design for these interventions, taking into account other related lifestyle factors, particularly exercise, also becomes evident. Finally, more studies are needed in order to understand how these factors influence each other, and the mechanisms underpinning the associations and the causality, so as to enhance the precision of advice and interventions.

## Figures and Tables

**Table 1:** Characteristics of the sample according to educational level and sex.

Variables	Third Grade			Sixth Grade			Total Sample		
	Females (*n* = 65)	Males (*n* = 73)	Total (*n* = 138)	Females (*n* = 86)	Males (*n* = 85)	Total (*n* = 171)	Females (*n* = 151)	Males (*n* = 158)	Total (*n* = 309)
***Child characteristics***									
Age (years), mean (SD)	8.3 (0.5)	8.3 (0.5)	8.3 (0.5)	11.4 (0.6)	11.5 (0.6)	11.4 (0.6)	10.1 (1.7)	10.0 (1.7)	10 (1.7)
Weight (kg), mean (SD)	32.2 (8.4)	32.7 (6.3)	32.4 (7.3)	45.3 (10.7)	45.6 (12.0)	45.5 (11.3)	39.6 (11.7)	39.6 (11.7)	39.6 (11.7)
Height (cm), mean (SD)	133.9 (6.4)	135.7 (6.4)	134.8 (6.4)	150.1 (8.7)	149.8 (9.6)	149.9 (9.1)	143 (11.2)	143.2 (10.9)	143.1 (11)
BMI (kg/m^2^), mean (SD)	17.8 (3.6)	17.7 (2.7)	17.7 (3.1)	20.1 (4.0)	20.1 (3.5)	20.1 (3.7)	19.1 (4.0)	19.0 (3.4)	19 (3.7)
BMI Categories, n (%) ^1^									
Underweight	6 (9.2)	5 (6.9)	11 (8.0)	10 (11.8)	4 (4.8)	14 (8.3)	16 (10.7)	9 (5.7)	25 (8.1)
Normal-weight	41 (63.1)	49 (67.1)	90 (65.2)	49 (57.7)	51 (60.7)	100 (59.2)	90 (60.0)	100 (63.7)	190 (61.9)
Overweight	12 (18.5)	14 (19.2)	26 (18.8)	21 (24.7)	24 (28.6)	45 (26.6)	33 (22.0)	38 (24.2)	71 (23.1)
Obesity	6 (9.2)	5 (6.9)	11 (8.0)	5 (5.9)	5 (6.0)	10 (5.9)	11 (7.3)	10 (6.4)	21 (6.8)
***Parents characteristic***									
Maternal age (years), mean (SD)	39.6 (5.0)	40.8 (5.2)	40.2 (5.1)	42.6 (5.0)	42.9 (5.2)	42.7 (5.1)	41.3 (5.2)	41.9 (5.3)	41.6 (5.3)
Paternal age (years), mean (SD)	42.6 (5.1)	43 (5.1)	42.8 (5.1)	45.1 (5.3)	45.4 (5.5)	45.3 (5.4)	44.0 (5.3)	44.3 (5.4)	44.2 (5.4)
Maternal education, n (%) ^1^									
Primary	8 (18.2)	7 (13.7)	15 (15.8)	14 (17.5)	14 (20.0)	28 (18.7)	22 (17.7)	21 (17.4)	43 (17.6)
Secondary/University	36 (81.8)	44 (86.3)	80 (84.2)	66 (82.5)	56 (80.0)	122 (81.3)	102 (82.3)	100 (82.6)	202 (82.4)
Paternal education, n (%) ^1^									
Primary	8 (19.1)	11 (21.6)	19 (20.4)	18 (23.7)	15 (21.4)	33 (22.6)	26 (22.0)	26 (21.5)	52 (21.8)
Secondary/University	34 (81.0)	40 (78.4)	74 (79.6)	58 (76.3)	55 (78.6)	113 (77.4)	92 (78.0)	95 (78.5)	187 (78.2)
***Lifestyle behaviors***									
Exercise practice outside school (days/week), mean (SD)	3.5 (1.9)	4.1 (1.7)	3.8 (1.8)	3.5 (1.8)	4.2 (2)	3.8 (1.9)	3.5 (1.8)	4.1 (1.8)	3.8 (1.9)
Attendance at the school canteen (days/week), mean (SD)	2.6 (2.4)	2.3 (2.4)	2.4 (2.4)	1.4 (2.2)	1.6 (2.2)	1.5 (2.2)	1.9 (2.3)	1.9 (2.3)	1.9 (2.3)
Sleeping habits (h/weekday), mean (SD)	9.9 (0.9)	9.8 (0.9)	9.8 (0.9)	9.3 (0.9)	9.3 (1.0)	9.3 (1.0)	9.5 (1.0)	9.5 (1.0)	9.5 (1.0)

BMI: body mass index. SD: standard deviation. ^1^ Differences in totals are due to missing values.

**Table 2:** Associations of KIDMED score and KIDMED items with weight status and lifestyle variables.

	BMI Categories		Weekly Days Exercise Practice Outside School		Weekly Days Attendance at the School Canteen		Sleep Time on Weekdays	
	OR (95% CI)	*p*-Value	OR (95% CI)	*p*-Value	OR (95% CI)	*p*-Value	OR (95% CI)	*p*-Value
**KIDMED Score Categories**	1.11 (0.66, 1.86)	0.7	1.17 (1.02, 1.33)	0.02	1.09 (0.98, 1.21)	0.1	1.18 (0.91, 1.54)	0.21
**KIDMED Items**								
Consumes a fruit or fruit juice every day	1.64 (0.72, 4.11)	0.26	1.31 (1.10, 1.57)	<0.01	1.17 (1.03, 1.36)	0.03	0.97 (0.71, 1.32)	0.84
Has a second piece of fruit every day	1.47 (0.90, 2.43)	0.13	1.10 (0.97, 1.25)	0.14	1.09 (0.90, 1.22)	0.09	1.15 (0.90, 1.48)	0.26
Has fresh or cooked vegetables regularly once a day	1.04 (0.63, 1.73)	0.9	1.07 (0.95, 1.22)	0.28	1.10 (1.00, 1.22)	0.06	1.58 (0.87, 2.85)	0.13
Has fresh or cooked vegetables more than once a day	1.58 (0.87, 2.85)	0.13	0.99 (0.87, 1.13)	0.93	1.11 (1.00, 1.23)	0.06	1.11 (0.84, 1.46)	0.47
Consumes fish regularly (at least 2–3/week)	0.95 (0.53, 1.73)	0.87	1.11 (0.95, 1.29)	0.19	1.17 (1.03, 1.34)	0.02	1.06 (0.78, 1.42)	0.72
Goes >1/week to a fast-food restaurant	0.95 (0.43, 1.95)	0.88	0.93 (0.77, 1.12)	0.47	0.97 (0.82, 1.12)	0.66	1.13 (0.79, 1.64)	0.50
Likes pulses and eats >1/week	0.59 (0.23, 1.60)	0.28	1.09 (0.91, 1.33)	0.36	1.02 (0.88, 1.19)	0.76	1.28 (0.88, 1.87)	0.19
Consumes pasta/rice every day (5 or more per week)	1.12 (0.61, 2.02)	0.71	1.05 (0.92, 1.20)	0.46	1.00 (0.90, 1.11)	0.95	0.98 (0.76, 1.28)	0.89
Has cereals or grains (bread, etc.) for breakfast	1.23 (0.60, 2.66)	0.59	1.24 (1.03, 1.51)	0.03	1.01 (0.87, 1.17)	0.93	1.02 (0.71, 1.46)	0.91
Consumes nuts regularly (at least 2–3/week)	1.43 (0.85, 2.41)	0.18	0.97 (0.85, 1.10)	0.58	1.04 (0.94, 1.16)	0.44	1.15 (0.89, 1.49)	0.30
Uses olive oil at home	0.68 (0.24, 2.03)	0.48	1.19 (0.90, 1.61)	0.23	1.03 (0.81, 1.34)	0.81	1.14 (0.66, 1.93)	0.62
Skips breakfast	1.03 (0.21, 4.15)	0.97	0.89 (0.67, 1.17)	0.42	1.06 (0.86, 1.31)	0.55	0.65 (0.39, 1.11)	0.11
Has a dairy product for breakfast (yoghurt, milk, etc)	4.03 (1.52, 12.97)	0.01	0.93 (0.79, 1.11)	0.42	1.06 (0.92, 1.23)	0.41	1.30 (0.90, 1.88)	0.16
Has commercially baked goods or pastries for breakfast	0.70 (0.39, 1.23)	0.23	0.91 (0.79, 1.05)	0.22	1.00 (0.89, 1.12)	0.96	0.92 (0.69, 1.22)	0.56
Consumes two yoghurts and/or some cheese (40g) daily	0.75 (0.41, 1.38)	0.36	1.01 (0.89, 1.14)	0.92	1.03 (0.93, 1.15)	0.53	1.02 (0.80, 1.31)	0.85
Consumes sweets and candy several times every day	0.96 (0.38, 2.25)	0.92	0.98 (0.79, 1.22)	0.87	1.09 (0.90, 1.31)	0.37	0.83 (0.55, 1.27)	0.39

Logistic regression analysis: Models were minimally adjusted as described in *Statistical analysis.* BMI: body mass index. OR: odds ratio. CI: confidence interval. KIDMED score was categorized by grouping the “low” and “medium” adherence categories, transforming it into a dichotomous variable. BMI categories were categorized by grouping the “underweight” and “normal-weight” and the “overweight” and “obesity” categories, transforming these into a dichotomous variable. The reference category for the independent variable, BMI, was “underweight/normal-weight”.

## Supplementary Materials

The following are available online at https://www.mdpi.com/article/10.3390/nu13051507/s1, Table S1: Results of the KIDMED test according to educational level and sex.
