# Supplementary material for: Adherence to the Mediterranean Diet in a School Population in the Principality of Asturias (Spain): Relationship with Physical Activity and Body Weight
Source: Nutrients. 2021 Apr 29;13(5):1507. doi: 10.3390/nu13051507 (PMC8145401; doi:10.3390/nu13051507)
Supplement: Supplementary file 1 [file nutrients-13-01507-s001.zip › nutrients-1179021-SI.pdf]

Table S1. Results of the KIDMED Test according to educational level and sex.

	Third grade			Sixth grade			Total sample		
	Females (*n* = 65)	Males (*n* = 73)	Total (*n* = 138)	Females (*n* = 86)	Males (*n* = 85)	Total (*n* = 171)	Females (n = 151)	Males (n = 158)	Total (*n* = 309)
**KIDMED Score, mean (SD)[1]**	7.9 (1.9)	7.3 (2.4)	7.6 (2.2)	7.2 (2.1)	7.9 (2.2)	7.6 (2.2)	7.5 (2.1)	7.6 (2.3)	7.5 (2.2)
**KIDMED Score, n(%)[2]**									
Low	0 (0)	4 (5.5)	4 (2.9)	6 (7)	3 (3.5)	9 (5.3)	6 (4)	7 (4.4)	13 (4.2)
Medium	30 (46.2)	33 (45.2)	63 (45.7)	36 (41.9)	29 (34.1)	65 (38)	66 (43.7)	62 (39.2)	128 (41.4)
Optimal	35 (53.9)	36 (49.3)	71 (51.5)	44 (51.2)	53 (62.4)	97 (56.7)	79 (52.3)	89 (56.3)	168 (54.4)
**KIDMED Items, n(%)[3]**									
Consumes a fruit or fruit juice every day	56 (86.2)	56 (76.7)	112 (81.2)	70 (81.4)	74 (87.1)	144 (84.2)	126 (83.4)	130 (82.3)	256 (82.9)
Has a second fruit every day	43 (66.2)	42 (57.5)	85 (61.6)	46 (54.1)	51 (60)	97 (57.1)	89 (59.3)	93 (58.9)	182 (58.9)
Has fresh or cooked vegetables regularly once a day	43 (66.2)	41 (56.2)	84 (60.9)	52 (60.5)	51 (60)	103 (60.2)	95 (62.9)	92 (58.2)	187 (60.5)
Has fresh or cooked vegetables more than once a day	26 (40)	21 (28.8)	47 (34.1)	19 (22.1)	32 (38.1)	51 (30)	45 (29.8)	53 (33.8)	98 (31.7)
Consumes fish regularly (at least 2-3/week)	48 (73.9)	61 (83.6)	109 (79)	64 (74.4)	61 (71.8)	125 (73.1)	112 (74.2)	122 (77.2)	234 (75.7)
Goes >1/week to a fast-food restaurant	5 (7.7)	12 (16.4)	17 (12.3)	12 (14)	9 (10.6)	21 (12.3)	17 (11.3)	21 (13.3)	38 (12.3)
Likes pulses and eats >1/week	53 (81.5)	59 (80.8)	112 (81.2)	77 (89.5)	82 (96.5)	159 (93)	130 (86.1)	141 (89.2)	271 (87.7)
Consumes pasta/rice every day (5 or more per week)	23 (35.4)	29 (39.7)	52 (37.7)	24 (27.9)	37 (43.5)	61 (35.7)	47 (31.1)	66 (41.8)	113 (36.6)
Has cereals or grains (bread. etc.) for breakfast	56 (86.2)	59 (80.8)	115 (83.3)	73 (85.9)	78 (91.8)	151 (88.8)	129 (86)	137 (86.7)	266 (86.1)
Consumes nuts regularly (at least 2-3/week)	34 (52.3)	41 (56.2)	75 (54.4)	39 (45.4)	44 (51.8)	83 (48.5)	73 (48.3)	85 (53.8)	158 (51.1)
Uses olive oil at home	61 (96.8)	69 (95.8)	130 (96.3)	78 (90.7)	82 (96.5)	160 (93.6)	139 (93.3)	151 (96.2)	290 (93.9)
Skips breakfast	5 (7.7)	3 (4.1)	8 (5.8)	6 (7)	4 (4.7)	10 (5.9)	11 (7.3)	7 (4.4)	18 (5.8)
Has a dairy product for breakfast (yoghurt. milk. etc)	54 (83.1)	61 (83.6)	115 (83.3)	70 (81.4)	75 (88.2)	145 (84.8)	124 (82.1)	136 (86.1)	260 (84.1)
Has commercially baked good or pastries for breakfast	19 (29.7)	28 (38.4)	47 (34.3)	18 (20.9)	25 (30.1)	43 (25.4)	37 (24.7)	53 (34)	90 (29.1)
Consumes two yoghurts and/or some cheese (40g) daily	45 (71.4)	40 (54.8)	85 (62.5)	46 (54.1)	54 (63.5)	100 (58.8)	91 (61.5)	94 (59.5)	185 (59.9)
Consumes sweets and candy several times every day	6 (9.4)	7 (10.1)	13 (9.8)	10 (11.8)	9 (10.7)	19 (11.2)	16 (10.7)	16 (10.5)	32 (10.4)

SD: standard deviation. [1]Results of the KIDMED test as a continuous variable. [2]Results of the KIDMED test as a categorical variable. [3] n(%) indicate the number of children who answered affirmatively to each item.
